# Biochemical changes in whole blood stored for transfusion at Bungoma County Referral Hospital, Kenya

**DOI:** 10.4102/ajlm.v9i1.1182

**Published:** 2020-12-21

**Authors:** Phidelis M. Marabi, Stanslaus Musyoki, Angela Amayo

**Affiliations:** 1 Department of Health, Bungoma County Referral Hospital, Bungoma, Kenya; 2 School of Health Sciences, Kisii University, Kisii, Kenya; 3 Department of Human Pathology, Faculty of Health Sciences, University of Nairobi, Nairobi, Kenya

**Keywords:** blood, transfusion, biochemical changes, storage, Kenya

## Abstract

**Background:**

During storage, transfusion blood may undergo a series of biochemical changes that could pose risks to patients when used. It is important therefore to monitor biochemical changes that may reduce survival or function of stored blood cells.

**Objective:**

This study assessed biochemical changes in whole blood stored for transfusion at Bungoma County Referral Hospital in the western region of Kenya between February 2019 and August 2019.

**Methods:**

A prospective study design involving 20 randomly selected donor blood units in citrate phosphate dextrose adenine anticoagulant was employed. Biochemical changes were evaluated for 35 days. Potassium and sodium levels were tested using the HumaLyte Plus5 analyser. Blood pH level was estimated using the Hanna pH meter.

**Results:**

At the end of the 35 days of storage under blood bank conditions, the mean potassium level significantly increased from 7.31 mmol/L at baseline to 20.14 mmol/L at week 5 (*p* < 0.0001), and the mean sodium level significantly decreased from 150.72 mmol/L at baseline to 121.56 mmol/L at week 5 (*p* < 0.0001). The pH level decreased insignificantly from 7.48 at baseline to 7.38 at the end of week 1 (*p* = 0.0757) but decreased significantly to 6.15 at the end of week 5 (*p <* 0.0001).

**Conclusion:**

Potassium increased and sodium concentrations decreased significantly from the first week of blood storage. The pH decreased significantly from the second week of storage. Therefore, aged blood should be avoided to circumvent potential adverse outcomes from biochemical changes and stored blood should be tested before use.

## Introduction

Biochemical variations take place during transfusion blood reservations, affecting their function.^1^ For many years, researchers have investigated the biochemical variations that take place in blood reserved before transfusions. More recently, clinical investigations have proposed that blood units reserved for extended periods (frequently detailed as 14–21 days) might be detrimental to the recipients, causing morbidity and mortality.^2^ Potassium is the prominent intracellular cation with sodium being the prominent extracellular cation. Maintenance of the circulation of potassium and sodium between the intracellular and the extracellular environments depends on a handful of homeostatic mechanisms. Literature discloses that potassium efflux, a renowned effect of prolonged red blood cell reservation, causes vesicle creation and may have a significant effect on the transfusion outcome.^3^ Studies have also shown a steady decrease in pH throughout the blood storage period which renders the cell membrane too rigid and predisposes the cells to lysis.^4^

Blood reservation improves access to blood for transfusions. However, questions arise regarding the toxicity and value of such blood reserved for extended periods. In various medical retrospective investigations, authors propose that transfusion of reserved blood may cause harm, particularly in severely ill patients such as patients with severe myocardial infarction conditions.^2^ They detected an elevated incidence of death, morbidity, infections, renal and lung breakdown, swelling, and thrombosis in patients who received extensively reserved blood compared to those who received fresh blood^5^; however, the causes of these remain unknown. These adverse effects may be attributed to the inability of red blood cells in reserved blood to transport oxygen as a result of the reduced concentration of adenosine triphosphate and 2, 3-diphosphoglycerate.^4^ Also, cytokines, enzymes, and ions such as potassium and calcium from white blood cells in stored blood may modify red blood cells and adversely affect transfused recipients. These modifications affect an assortment of membrane molecules involved with adhesion, oxygen delivery and complement modulation.^6^ The storage of blood for extended periods can potentially result in the manipulation of the membrane exterior of red blood cells, and consequently their biological functions.^6^

According to records of Bungoma County Referral Hospital in Bungoma, Kenya, the transfusion rate averages 300 transfusions monthly and there has been an increase in blood reactions in the patients that receive a blood transfusion. A monthly average of 2% – 3% of patients react to transfused blood, especially aged blood, which is blood that has been stored for over 20 days. Despite the high levels of post-reaction after transfusions in Bungoma County Referral Hospital and the risk of toxicity to patients due to leakage of blood chemicals, the biochemical changes in blood stored for transfusions have not been studied and remain unknown. Therefore, this study aimed to assess the biochemical changes in whole blood stored for transfusions at Bungoma County Referral Hospital.

## Materials and methods

### Ethical considerations

The study was cleared by the Ethical Committee of Jaramogi Oginga Teaching and Referral Hospital (#ERC.IB/VOL.1/454) and authority to carry out the research was issued by the National Commission for Science, Technology and Innovation (#NACOSTI/P/19/32125/27143). Written informed consent was obtained from each donor after a brief explanation of what the study is about. Participation in the study was voluntary. Donor details were kept confidential by the exclusion of all forms of identification on the data collection tool, and filled data tools were stored physically under lock and digitally with restricted password access on a computer. The donors were informed that their blood units, if selected for the study, were not transfused to patients.

### Study area

The study was carried out between February 2019 and August 2019 at Bungoma County Referral Hospital, which is located in the western region of Kenya. This was a good study area because adverse reactions among the patients who receive a blood transfusion was common. The hospital also has an accredited laboratory that is well equipped with biochemistry equipment required for the study. The hospital has a blood donation centre which collects on average 600 blood units monthly.

### Study design

This was a prospective study involving the collection of blood from healthy volunteers into blood bags containing citrate phosphate dextrose adenine anticoagulant. All donated blood samples were reserved at temperatures between 2 °C and 6 °C and analysed at baseline and then at the ends of week one, two, three, four and five. The whole units of blood were used in the study to ensure that all storage conditions for blood meant for transfusions were met.

### Study population

The sample size for the research was determined by Yamane Taro’s formulae for a finite population.^7^ A sample size of 20 blood units was adopted for the study. A simple random sampling technique was used in this study where every 10th sample was selected for the study in order to eliminate bias.

### Data collection

#### Sample collection and analysis

All blood units were collected according to blood transfusion donor guidelines as described by the World Health Organization.^8^ At baseline, samples were immediately separated from blood units collected from the volunteer donors to test for potassium, podium and pH levels. Blood units were then stored at blood bank conditions of 2 °C – 6 °C for 35 days with intermittent sampling at 7-day intervals to test for potassium and sodium levels. Potassium and sodium levels were tested using a HumaLyte Plus5 analyser (Human Diagnostics Worldwide, Wiesbaden, Germany) while pH was estimated using a Hanna pH meter (Scientific Instrumentation, Woonsocket, Rhode Island, United States). Briefly, the plasma supernatant was aseptically transferred from blood units to plain test tubes and brought to the right temperature as per the manufacturer’s instructions before being fed into the machine for automated analysis of potassium and sodium. The instrument measures the electrode potentials, and the data are processed by the microprocessor to obtain the concentration of a given ion. The normal range for potassium concentration is between 3.5 mmol/L and 5.5 mmol/L and between 145 mmol/L and 155 mmol/L for sodium concentration. Aliquots of the whole blood units were taken for pH determination at the study points using the automated Hanna pH meter, with results displayed on the screen. The normal range for blood pH is 7.35–7.45. All laboratory results were recorded on a standard data collection form developed for this study.

#### Quality assurance of the data

To ensure the quality of data collected, pre-donation requirements (such as ensuring that donors had a haemoglobin level of 12.5 g/dL and above, weighed 50 kg and above, had no transfusion in the past 12 months, had not donated blood in the past 3 months, had no history and signs of malignancy, had no signs and symptoms of sickle cell disease, had no signs and symptoms of polycythaemia Rubra Vera and had no history of haemophilia and other coagulation disorders) were followed and only blood units that met these criteria were used. A qualified phlebotomist collected the blood samples, ensuring that the right quantity of between 450 mL and 500 mL was collected. The pints were transported and stored as per blood transfusion guidelines at 2 °C – 6 °C. Sample aliquots at the various study points were brought to optimum temperature as per the manufacturer’s instructions before testing. Samples were analysed in duplicate for each sample and an average computed to ensure accuracy. Both external and internal quality controls for pH, sodium and potassium tests were verified and ensured during the study. Recorded results were verified by a second analyst to ensure accuracy.

The laboratory is enrolled in the Human Quality Assessment Services External Quality Assurance Scheme for chemistry scope including pH, sodium and potassium. The chemistry scope is also accredited by the Kenya Accreditation Services which further assured the quality of data collected.

### Data management and analysis

The data were stored in Microsoft Excel (Microsoft Corporation, Redmond, Washington, United States). Data analysis was done using the Statistical Package for the Social Sciences (SPSS V.23) (IBM Corporation, Chicago, Illinois, United States). Descriptive statistics (frequencies, mean and standard deviation) were used to describe the data. The trends of the biochemical changes were shown using line plots. Analysis of variance was used to establish if there were significant biochemical changes in transfusion blood at baseline and each study point compared to normal reference intervals for 35 days of storage. Findings were considered significant at a *p*-value less than 0.05. Tukey’s honest significant difference test was used to collate all feasible pairs of means.

## Results

**FIGURE 1:**
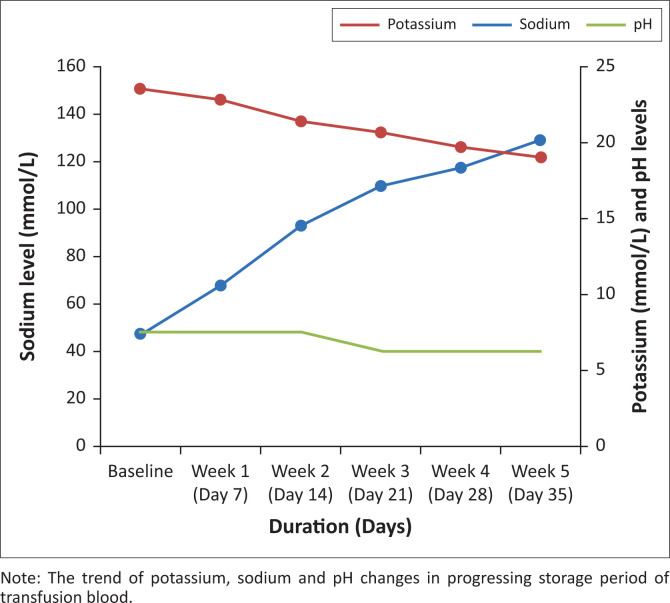
Potassium levels demonstrated an increasing trend, while sodium levels and pH demonstrated a decreasing trend as storage period progressed, February 2019 to August 2019.

Potassium (K+) levels increased progressively from 7.31 mmol/L to 20.14 mmol/L, while sodium (Na+) levels and pH respectively demonstrated corresponding decrease from 150.72 mmol/L to 121.56 mmol/L and from 7.48 to 6.15 as storage period progressed (Figure 1).

This study observed significant variance in potassium levels at baseline compared to the normal reference interval median of 4.5 mmol/L. Potassium levels also increased significantly throughout the blood storage period compared to the baseline mean of 7.31 mmol/L (Table 1). There was no significant variance in sodium concentrations at the baseline compared to the normal reference interval median of 150 mmol/L; however, a significant decrease in sodium concentration was observed throughout the blood storage period compared to the baseline. pH levels demonstrated no significant variance at baseline compared to the normal reference interval median of 7.4 and no significant variance at the end of week 1 (Day 7) compared to baseline. pH levels at weeks 2, 3, 4 and 5 however decreased significantly from 7.31 at baseline (Table 1).

**TABLE 1:** Biochemical changes in blood throughout the blood storage period at Bungoma County Referral Hospital, Kenya, February 2019 to August 2019.

Parameters	Baseline (Day 0)†	Week 1 (Day 7)‡	Week 2 (Day 14)‡	Week 3 (Day 21)‡	Week 4 (Day 28)‡	Week 5 (Day 35)‡
**Potassium (K+)**						
Mean	7.31 mmol/L	10.59 mmol/L	14.57 mmol/L	17.15 mmol/L	18.33 mmol/L	20.14 mmol/L
*F*	128.62	54.06	176.82	219.81	222.90	296.40
*p*	< 0.0001	< 0.0001	< 0.0001	< 0.0001	< 0.0001	< 0.0001
**Sodium (Na+)**						
Mean	150.72 mmol/L	146.16 mmol/L	137.21 mmol/L	132.53 mmol/L	126.26 mmol/L	121.56 mmol/L
*F*	1.62	49.04	300.75	225.12	278.72	400.18
*p*	0.2102	< 0.0001	< 0.0001	< 0.0001	< 0.0001	< 0.0001
**pH**						
Mean	7.48	7.38	7.26	6.45	6.27	6.15
*F*	4.41	33	15.51	279.82	446.44	570.09
*p*	0.4826	0.0757	0.0003	< 0.0001	< 0.0001	< 0.0001

*F*, A ratio of two variances.

† , Compared to the normal reference interval median.

‡ , Compared to baseline.

## Discussion

The current study sought to evaluate the biochemical changes in whole blood for transfusion stored at blood bank conditions. At the end of the 35 days of blood storage at blood bank conditions, the potassium levels significantly increased while sodium levels significantly decreased over time. On the other hand, the pH levels significantly decreased over time; however, the change was insignificant at week 2 of storage. Potassium levels had a significant variation from normal reference at the baseline while sodium concentrations and pH had insignificant variance from normal reference intervals. Regardless of the advantages of blood transfusion in clinical care (such as to treat anaemia, prepare patients for a surgical process, and limit manifestations of blood depletion), the procedure may cause harm to patients.^9^ There are various deleterious after-effects of blood transfusion that could occur despite cautious laboratory methods in handling and cross-matching the donors and the recipient. The undesired effects may be attributed to changes in the usual micro-environment or biochemistry of the blood cells in the course of blood storage.

The present study has found that there are biochemical variations in blood during the 35 days storage period. Potassium ions increased significantly after one week and further increased significantly through to 35 days of storage. This result compares with previous studies in Portugal,^10^ Uganda,^9^ India^11^ and Nigeria^12^ which demonstrated that potassium significantly increased throughout the storage period.

The current study observed an increase of plasma potassium level to 10.59 mmol/L within the first week. The increase continued to 20.14 mmol/L at the end of the fifth week which was equivalent to an average of 12.83 mmol/L rise after 35 days of storage. This indicates that blood transfusion after the first week of storage may not be safe for patients due to the potassium levels. In the course of blood reservations, there is a steady but continuous efflux of potassium ions from cells into the encircling plasma.^12^ During critical kidney disorder, even minute amounts of potassium ion variations are potentially threatening, and relatively fresh or washed red cells are recommended. Potassium ions efflux is recognised as a secondary metabolic change that occurs due to cooling.^12^ The rapid effusion of potassium ions from cells into encircling plasma possibly accounts for the radical elevation in potassium ion concentration in this study. Based on the findings of the present study, monitoring of potassium ion concentration during storage of blood for transfusion is recommended to improve blood safety.

The current study observed that a decrease in sodium levels was significant after 2 weeks (14 days) and further decreased significantly through to 35 days of storage. This result compares with previous studies in India, Portugal and Ghana which demonstrated that sodium levels decreased throughout the storage period.^10,11,13^ Our results, however, contrast a study done in India which demonstrated that sodium did not show any significant changes due to storage time^14^ as well as another study done in Nigeria in which reservation period was not established to influence sodium concentration.^12^

The reference range for plasma sodium level is 145 mmol/L – 155 mmol/L. The findings of this study indicate that within the first week of storage, sodium levels in blood could fall below the normal range. Blood stored at blood bank conditions slows down cellular metabolism and energy demand which permits blood to be reserved for 35–42 days. This makes the sodium-potassium pump defective and, as a result, permits potassium ions to depart the cell and sodium ions to move into the cells through the semipermeable membrane.^10^ Therefore, these significant changes may affect patients that may be affected by low levels of sodium such as an increased chance of such a recipient being prone to oedema especially in patients with low sodium intake or those experiencing diarrhoea.^15^ In light of the findings from the present study, monitoring of sodium ion concentration during storage of blood for transfusion with the goal to improve blood safety is recommended.

This study reported a significant pH decrease after 14 days of storage which further decreased significantly through to 35 days of storage. This result compares with previous studies in Uganda, India and Ghana which demonstrated that the pH of reserved blood lessened during the storage period.^9,10^ The reference range for blood pH is 7.35–7.45; this study reported a decrease in blood pH from 7.48 to 6.15 which was equivalent to an average drop of 1.33 after 35 days of storage. This indicates that the pH decreased far below the normal range by the end of 35 days. According to Oyet et al.,^9^ the increase in protons due to accumulation of lactate after 14 days of storage causes the pH level to decrease and subsequently changes glycolytic metabolism. The elevated lactate level with the reciprocal descent in pH exerts ravaging consequences on blood receivers, principally those who may be given several units of blood in a short period. This diminishes blood potency and predisposes blood receivers to undesired transfusion-associated morbidity and mortality.^9^ The depletion of diphosphoglycerate and diminished glycolytic action are also linked to lessening pH.^12^ This study recommends that transfusion of blood reserved for more than 1 week should be exercised with caution. Taking into account the findings from the present study, pH level monitoring during blood storage especially after the first week of storage with the focus on improving blood safety is recommended.

Overall, together with other parameters, biochemical changes in stored blood for blood transfusion should be properly monitored especially in consideration of the patient to be transfused. Because of the increase in potassium level with storage, caution is required to ascertain the storage duration of blood to be transfused, volume to be transfused at a time, and rate of transfusion to decrease hyperkalaemia-linked blood transfusion problems. Transfusing hyperkalaemic blood, however, may have little effect if the recipient’s kidneys are working normally and the sodium-potassium adenosine triphosphate pump is also working efficiently. However, this is a significant concern in recipients with renal failure.

### Recommendations

The study recommends the monitoring of biochemical changes and the use of fresh blood to avoid the negative effect of changes in stored blood especially to patients who suffer from renal failure and myocardial infarction.

### Limitations

In the current study, other biochemical parameters such as iron, lactate, calcium, and so on, which could have an impact on the viability of blood were not assessed.

### Conclusion

This study showed that variations in biochemical parameters occur during the storage of blood despite the use of adequate storage conditions, and may cause risks to patients during transfusion.
